# Thyroid function and risk of type 2 diabetes: a population-based prospective cohort study

**DOI:** 10.1186/s12916-016-0693-4

**Published:** 2016-09-30

**Authors:** Layal Chaker, Symen Ligthart, Tim I. M. Korevaar, Albert Hofman, Oscar H. Franco, Robin P. Peeters, Abbas Dehghan

**Affiliations:** 1 Rotterdam Thyroid Center, Erasmus University Medical Center, Rotterdam, The Netherlands; 2 Department of Internal Medicine, Erasmus University Medical Center, Rotterdam, The Netherlands; 3 Department of Epidemiology, Erasmus University Medical Center, Room NA-2828, 3000CA Rotterdam, The Netherlands; 4 Department of Epidemiology, Harvard T.H. Chan School of Public Health, Boston, MA USA

**Keywords:** Type 2 diabetes, Thyroid hormone, Thyroid function, Diabetes, Prediabetes

## Abstract

**Background:**

The association of thyroid function with risk of type 2 diabetes remains elusive. We aimed to investigate the association of thyroid function with incident diabetes and progression from prediabetes to diabetes in a population-based prospective cohort study.

**Methods:**

We included 8452 participants (mean age 65 years) with thyroid function measurement, defined by thyroid-stimulating hormone (TSH) and free thyroxine (FT4), and longitudinal assessment of diabetes incidence. Cox-models were used to investigate the association of TSH and FT4 with diabetes and progression from prediabetes to diabetes. Multivariable models were adjusted for age, sex, high-density lipoprotein cholesterol, and glucose at baseline, amongst others.

**Results:**

During a mean follow-up of 7.9 years, 798 diabetes cases occurred. Higher TSH levels were associated with a higher diabetes risk (hazard ratio [HR] 1.13; 95 % confidence interval [CI], 1.08–1.18, per logTSH), even within the reference range of thyroid function (HR 1.24; 95 % CI, 1.06–1.45). Higher FT4 levels were associated with a lower diabetes risk amongst all participants (HR 0.96; 95 % CI, 0.93–0.99, per 1 pmol/L) and in participants within the reference range of thyroid function (HR 0.96; 95 % CI, 0.92–0.99). The risk of progression from prediabetes to diabetes was higher with low-normal thyroid function (HR 1.32; 95 % CI, 1.06–1.64 for TSH and HR 0.91; 95 % CI, 0.86–0.97 for FT4). Absolute risk of developing diabetes type 2 in participants with prediabetes decreased from 35 % to almost 15 % with higher FT4 levels within the normal range.

**Conclusions:**

Low and low-normal thyroid function are risk factors for incident diabetes, especially in individuals with prediabetes. Future studies should investigate whether screening for and treatment of (subclinical) hypothyroidism is beneficial in subjects at risk of developing diabetes.

**Electronic supplementary material:**

The online version of this article (doi:10.1186/s12916-016-0693-4) contains supplementary material, which is available to authorized users.

## Background

Diabetes mellitus and thyroid disease are the two most common endocrine disorders, often co-existing in patients [1]. The role of auto-immunity has been well-recognized in the link between auto-immune thyroid disease and type 1 diabetes mellitus [2]. A relation between thyroid dysfunction and type 2 diabetes mellitus has also been suggested, but the possible underlying mechanisms and drivers show complex interactions [3].

Thyroid hormone is a major regulator of metabolism and energy expenditure, is directly involved in the control of insulin secretion and glucose homeostasis [3, 4], and has been shown to preserve beta-cell viability and proliferation [5, 6]. Hyperthyroid individuals have an increased insulin secretion [7] and higher free triiodothyronine levels are specifically associated with improved insulin secretion in individuals with prediabetes [8]. However, the deleterious effect of thyrotoxicosis on glucose metabolism has also been recognized for decades [9]. Excess thyroid hormone (i.e. hyperthyroidism) causes increased liver gluconeogenesis and peripheral insulin resistance and is associated with glucose intolerance [10–13]. Interestingly, lack of thyroid hormone is also associated with a decrease in peripheral insulin sensitivity and glucose intolerance [14] and treatment of hypothyroidism has been shown to improve insulin sensitivity [14, 15].

There are several cross-sectional reports on the association between thyroid dysfunction and diabetes, albeit with conflicting results, with some studies reporting an association between hyperthyroidism and type 2 diabetes, while others report instead an association between hypothyroidism and diabetes. Further, one of the most recent and largest cross-sectional studies reports no association between thyroid dysfunction and type 2 diabetes [16]. However, cross-sectional studies have several limitations, including lack of assessment of temporality. Only few studies have investigated the association of thyroid function with incidence of diabetes prospectively and all were register-based studies, again reporting conflicting results [17–19]. As a consequence, there is no consensus regarding whether patients with thyroid dysfunction should be screened for diabetes. To date, there are no prospective population-based cohort studies investigating the association across the full range of thyroid function, including the normal range, with the risk of diabetes. Therefore, we aimed to investigate the association of thyroid function with the incidence of type 2 diabetes and the progression from prediabetes to diabetes in the Rotterdam Study, a large prospective population-based cohort study.

## Methods

### The Rotterdam Study

The Rotterdam Study is a prospective population-based cohort study that investigates the determinants and occurrence of age-related diseases in Ommoord, Rotterdam, the Netherlands. The aims and design of the Rotterdam Study have been described in detail elsewhere [20]. The Rotterdam Study consists of three independent cohorts: RS Cohort I (RSI), including 7983 participants aged ≥ 55 years (baseline 1990–1993), RS Cohort II (RSII), including 3011 participants aged ≥ 55 years (baseline 2000–2001), and RS Cohort III (RSIII), including 3932 participants aged ≥ 45 years (baseline 2006–2008).

The Rotterdam Study has been approved by the medical ethics committee according to the Population Screening Act: Rotterdam Study, executed by the Ministry of Health, Welfare and Sports of the Netherlands.

### Study population

We selected data from participants from the third visit of the first cohort (1997–1999, *n* = 4797) and the first visit of the second (2000–2001, *n* = 3011) and third (2006–2008, *n* = 3932) cohorts, if thyroid-stimulating hormone (TSH) or free thyroxine (FT4) measurements, which were performed in a random set of participants, and information on diabetes were available. All participants in the present analysis provided written informed consent to participate and to obtain information from their treating physician. All study participants were followed up from the day of baseline laboratory testing to date of onset of diabetes, to death, or to January 1, 2012, whichever came first.

### Assessment of thyroid function

Thyroid function was measured using the same methods and assay for all three cohorts, and samples were collected between 1997 and 2008, depending on the cohort. TSH and FT4 measurements were performed in serum samples stored at –80 °C (electrochemiluminescence immunoassay for thyroxine and thyrotropin, “ECLIA”, Roche). We determined cut-off values for the reference range of TSH as 0.4–4.0 mIU/L and for FT4 as 11–25 pmol/L (0.86–1.94 ng/dL) according to guidelines as well as our previous studies [21]. Thyroid peroxidase antibody (TPOAb) levels greater than 35 kU/mL were regarded as positive, as recommended by the assay manufacturer (electrochemiluminescence immunoassay for thyroid peroxidase antibodies, “ECLIA”, Roche).

### Ascertainment of prediabetes and type 2 diabetes

At baseline and during follow-up, cases of prediabetes and type 2 diabetes were ascertained through active follow-up using general practitioners’ records, hospital discharge letters, and serum glucose measurements from Rotterdam Study visits, which take place approximately every 4 years [22]. Normoglycemia, prediabetes, and diabetes were defined according to recent WHO guidelines [23]; normoglycemia was defined as a fasting serum glucose < 6.0 mmol/L; prediabetes was defined as a fasting serum glucose > 6.0 mmol/L and < 7.0 mmol/L or a non-fasting serum glucose > 7.7 mmol/L and < 11.1 mmol/L (when fasting samples were absent); and type 2 diabetes was defined as a fasting serum glucose ≥ 7.0 mmol/L, a non-fasting serum glucose ≥ 11.1 mmol/L (when fasting samples were absent), or the use of blood glucose lowering medication. Information regarding the use of blood glucose lowering medication was derived from both structured home interviews and linkage to pharmacy records. At baseline, more than 95 % of the Rotterdam Study population was covered by the pharmacies in the study area. All potential events of type 2 diabetes were independently adjudicated by two study physicians. In case of disagreement, consensus was sought with an endocrinologist [22].

### Baseline measurements

Body mass index was calculated as body mass (kg) divided by the square of the body height (m). Serum HDL cholesterol and glucose were measured using standard laboratory techniques. Information on tobacco smoking was derived from baseline questionnaires. Systolic and diastolic blood pressure was calculated as the average of two consecutive measurements. Insulin was measured using an immunoassay (electrochemiluminescence immunoassay “ECLIA”, Roche). Over 95 % of participants were in a fasting state when blood was drawn at the Rotterdam Study center visit. Information on medication use was obtained from questionnaires in combination with pharmacy records. Thyroid medication, including thyroid hormone replacement therapy, was prescribed by participant’s own GP or specialist and within the context of regular treatment and blinded to measurements of the Rotterdam Study.

### Statistical methods

We used Cox-proportional hazards models to assess the association of TSH or FT4 with incident diabetes. We also assessed the association of thyroid function measurements and incident diabetes in participants with prediabetes separately. We first conducted these analyses in all included participants and then only in those with normal TSH and FT4 values, after excluding levothyroxine users. The primary model, model 1, was adjusted for age, sex, cohort, fasting glucose, and tobacco smoking. Model 2 was additionally adjusted for possible confounders or intermediate factors, including fasting serum insulin, systolic blood pressure, diastolic blood pressure, use of blood pressure lowering medication (diuretics, anti-adrenergic agents, β blockers, calcium channel blockers, and RAAS inhibitors), high-density lipoprotein (HDL) cholesterol and body mass index (BMI). Adjusting for both BMI and waist circumference showed multicollinearity in the model, with BMI providing the best model fit. Additionally adjusting for waist circumference next to BMI did not provide meaningful changes in the risk estimates and therefore waist circumference was omitted from the model. Furthermore, we assessed the association of TSH and FT4 tertiles in the normal reference range with progression from prediabetes to diabetes and calculated absolute risk estimates for the tertiles, using the covariates of the multivariable model. We performed the following sensitivity analyses: (1) excluding participants using levothyroxine at baseline, (2) excluding participants using thyroid function altering medication, including levothyroxine, anti-thyroid drugs (e.g., thiamazole), amiodarone, and corticosteroids at baseline and follow-up, and (3) additionally excluding participants with TSH and FT4 values outside the normal range. We stratified by possible effect modifiers, including age categories (cut-off of 65 years) and sex. The natural logarithm of TSH was used for the continuous models and results are presented per doubling of TSH on average. The proportional hazards assumption was assessed by performing Schoenfeld tests and plots and was met for all analyses. There was no departure from linearity as assessed by restricted cubic splines or adding quadratic terms of TSH, FT4, or age to the model. Reporting of the results is according to the STROBE statement.

## Results

**Fig. 1:**
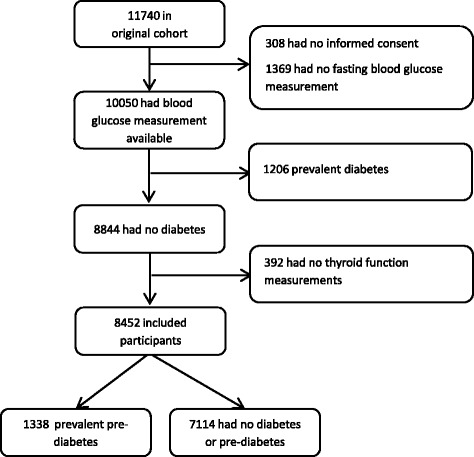
Participant selection

We included a total of 8452 participants with thyroid function measurements and who were free of diabetes at baseline (Fig. 1). The mean age of the included participants was 64.9 years and 58 % were female. Baseline characteristics are shown in Table 1. During a mean follow-up of 7.9 years (standard deviation 4.0 years), 798 individuals developed diabetes (IR 12 per 1000 person-years). Completeness of follow-up was 99.4 % [24].

**Table 1:** Baseline characteristics of included participants

Variable	Mean (SD)*
Number of individuals in the study	8452
Age, in years	64.6 (9.7)
Female, *n* (%)	4899 (58.0)
BMI, kg/m^2^	26.5 (4.05)
Total cholesterol, mmol/L	5.76 (1.01)
HDL cholesterol, mmol/L	1.43 (0.41)
Smoking, *n* (%)	
Current	1742 (20.6)
Former	4020 (47.6)
Never	2691 (31.8)
Systolic blood pressure, mmHg	139 (21)
Diastolic blood pressure, mmHg	79 (11)
Antihypertensive medication use, *n* (%)	1881 (22.3)
TSH, median (IQR)	1.91 (1.29–2.76)
FT4, pmol/L	15.7 (2.32)
TPOAb positivity, *n* (%)	1119 (13.2)
Levothyroxine use, *n* (%)	233 (2.8)

*unless specified otherwise

TPOAb levels > 35 kU/mL were regarded as positive

*BMI* body mass index, *IQR* interquartile range, *FT4* free thyroxine, *SD* standard deviation, *TPOAb* thyroid peroxidase antibodies, *TSH* thyroid-stimulating hormone, *n* number

### Thyroid function and incident diabetes

The associated risk of developing diabetes was 1.09 times higher for every doubling of TSH levels mIU/L (95 % confidence interval [CI], 1.06–1.12; Table 2). Within the normal range, the risk of diabetes was 1.16 times higher with higher TSH levels. In model 2, this association attenuated slightly (hazard ratio [HR] 1.06; 95 % CI, 1.00–1.13, Table 2). In the most adjusted model (model 2), higher FT4 levels were associated with a decreased risk of diabetes (HR 0.96; 95 % CI, 0.93–0.99), also within the normal range (HR 0.94; 95 % CI, 0.90–0.98).

**Table 2:** Association between thyroid function and the risk of incident prediabetes and diabetes

Thyroid function measurements	HR (95 % CI) Model 1	HR (95 % CI) Model 2	Incident cases	Total participants
Incident Diabetes				
Full range of measurement				
TSH mIU/L	1.09 (1.06–1.12)	1.06 (1.00–1.13)	798	8447
Free T4 pmol/L	0.96 (0.93–0.99)	0.96 (0.93–0.99)	797	8446
Normal TSH and FT4 values				
TSH mIU/L	1.16 (1.04–1.30)	1.14 (1.02–1.27)	685	7188
Free T4 pmol/L	0.96 (0.92–0.99)	0.94 (0.90–0.98)	685	7188
Progression from prediabetes to diabetes				
Full range of measurement				
TSH mIU/L	1.17 (1.07–1.27)	1.13 (1.03–1.24)	412	1337
Free T4 pmol/L	0.92 (0.89–0.97)	0.93 (0.89–0.98)	411	1336
Normal TSH and FT4 values				
TSH mIU/L	1.26 (1.08–1.47)	1.21 (1.04–1.41)	358	1137
Free T4 pmol/L	0.90 (0.85–0.95)	0.91 (0.86–0.97)	358	1137

Model 1: adjusted for sex, age, smoking, fasting serum glucose levels and cohort

Model 2: adjusted for sex, age, smoking, cohort, fasting serum glucose levels, fasting serum insulin measurements, systolic blood pressure, diastolic blood pressure, blood pressure lowering medication, HDL cholesterol, and body mass index

Normal range of TSH is defined by 0.4–4.0 mIU/L and normal range FT4 is defined by 11–25 pmol/L and participants not using levothyroxine

Results are presented as HR per doubling of TSH on average and per one increase in pmol/L of FT4

*CI* confidence interval, *FT4* free thyroxine, *HR* hazard ratio, *TSH* thyroid-stimulating hormone

Sensitivity analyses did not change risk estimates meaningfully (Additional file 1: Table S1). Stratifying the analyses by age category or sex did not show effect modification for incident diabetes (*P* for interaction > 0.05 for all).

### Thyroid function and progression of prediabetes to diabetes

**Fig. 2:**
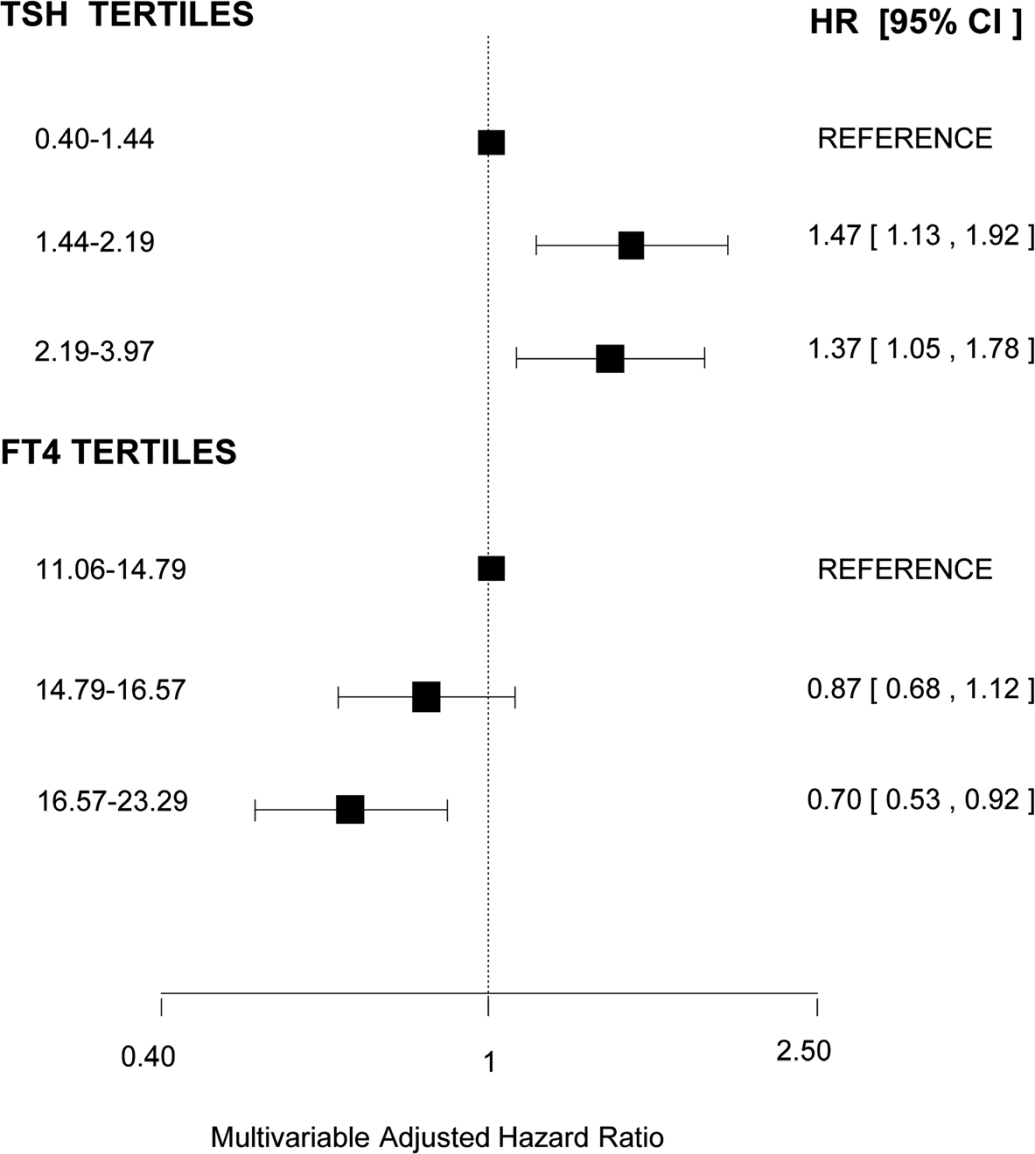
Association of thyroid-stimulating hormone (TSH) and free thyroxine (FT4) levels in tertiles within the normal range and incident diabetes in individuals with prediabetes. The normal range of TSH was defined as 0.4–4.0 mIU/L and of FT4 as 11–25 pmol/L (Conversion 1 pmol/L = 0.0777 ng/dL), thyroid hormone medication users were excluded. The analyses were adjusted for sex, age, smoking, cohort, fasting glucose, serum insulin measurements, systolic blood pressure, diastolic blood pressure, blood pressure lowering medication, cholesterol, and body mass index. *AF* atrial fibrillation, *HR* hazard ratio, *CI* confidence interval

**Fig. 3:**
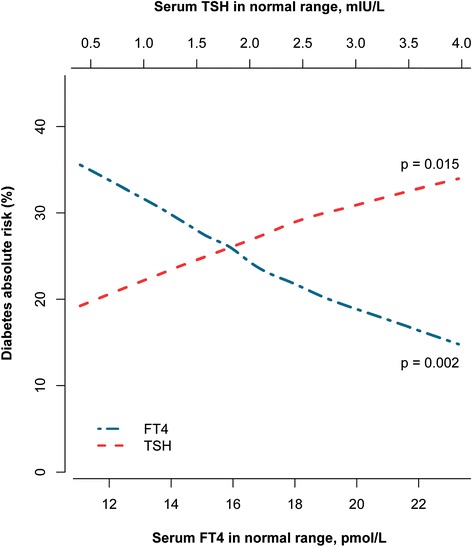
The 7-year absolute risk of progression from prediabetes to type 2 diabetes is plotted against thyroid-stimulating hormone (TSH) and free thyroxine (FT4) values within the normal range. These analyses are adjusted for sex, age, smoking, cohort, fasting serum glucose levels, fasting serum insulin measurements, systolic blood pressure, diastolic blood pressure, blood pressure lowering medication, high-density lipoprotein cholesterol, and body mass index

In participants with prediabetes, the associated risk of developing diabetes was 1.13 times higher for every doubling of TSH levels (95 % CI, 1.03–1.24; Table 2). The risk of incident diabetes in participants with prediabetes was 0.93 times lower with each 1 pmol/L increase of FT4 (95 % CI, 0.89–0.98). In the normal range, the risk of developing diabetes was 1.44 times higher (95 % CI, 1.13–1.93) when comparing the highest to the lowest tertile of TSH in the normal range in model 1 (Additional file 2: Table S2). This corresponds to an absolute risk difference of 8.5 % for a follow-up of 7 years. Comparing the highest to the lowest tertile for FT4, the HR for developing diabetes in individuals with prediabetes was 0.63 (95 % CI, 0.48–0.82; Additional file 2: Table S2). Additionally adjusting analyses for TPOAb positivity did not change risk estimates meaningfully (data not shown). This corresponds to a 1.59 times higher risk and an absolute risk difference of 9.6 % of progression to diabetes when comparing the lowest to the highest tertile of FT4 (Additional file 2: Table S2). These associations attenuated only slightly in model 2 (Fig. 2, Additional file 2: Table S2). Absolute risk of diabetes type 2 in participants with prediabetes decreased from 35 % to almost 15 % with higher FT4 levels within the normal range (Fig. 3).

## Discussion

To our knowledge, this is the first prospective population-based cohort study describing the relation between thyroid function within the normal range and the risk of diabetes and progression from prediabetes and type 2 diabetes. Higher TSH levels and lower FT4 levels are associated with an increased risk of diabetes and progression from prediabetes to diabetes.

There are no other studies addressing the relation between diabetes and thyroid function in the euthyroid range or in individuals with prediabetes. Even though there are many cross-sectional reports studying the prevalence of diabetes and thyroid dysfunction, only few have investigated the association of thyroid function with the occurrence of diabetes and all were register-based studies. Our results are in contrast to a Danish nationwide registry study by Brandt et al. [17] that reported an increased risk of diabetes in hyperthyroid individuals, whereas we did not find an increased risk of diabetes with higher thyroid function. However, there are several factors that could explain these differences, including variance in the mean age and possible iodine status of the studied population. Most importantly, the study by Brandt et al. [17] did not include laboratory measurements of thyroid function and therefore misclassification of the diagnosis of hyperthyroidism could have occurred. Further, they did not provide estimates in the euthyroid range of thyroid function. Two other register-based studies report an increased risk of diabetes in hypothyroid individuals [18, 19] and our results are largely in line as we find an increased risk of diabetes in lower thyroid function.

There are several pathways that may explain the observed relation between low and low-normal thyroid function and the risk of diabetes. Overt and subclinical hypothyroidism are associated with a decreased insulin sensitivity and glucose tolerance, partially due to a decreased ability of insulin to increase glucose utilization mainly in muscle [14, 25]. Other mechanisms, such as downregulation of plasma membrane glucose transporters and direct effects on insulin degradation, have also been described [26–28]. Treatment of hypothyroidism has been shown to restore insulin sensitivity and the secretion of glucoregulatory hormones [15]. Furthermore, hypothyroidism is associated with several components of the metabolic syndrome and could therefore indirectly relate to the increased risk of diabetes [29]. However, in our analyses, adjusting for several cardiovascular risk factors and components of the metabolic syndrome did not shift risk estimates towards the null. Additionally, excluding participants using thyroid hormone replacement therapy at baseline only slightly altered the results. Even though overt hyperthyroidism is also associated with insulin resistance, our data show that high and high-normal thyroid function are protective against the development of or progression to diabetes. It could be that insulin resistance in hyperthyroid patients is counterbalanced by other mechanisms associated with prolonged thyroid hormone excess, such as improved beta-cell function and increased insulin secretion [6]. However, the exact pathophysiological mechanisms through which thyroid function could affect diabetes risk in the general population remain to be determined.

The clinical importance of these findings could be several. First of all, the association of thyroid function with development from prediabetes to diabetes is prominent. Thus, individuals with a low-normal thyroid function, which includes a large proportion of the population, are at an even higher risk of progression from prediabetes to diabetes. Secondly, with ageing and increasingly obese populations, there is need for better screening and prevention options for diabetes [30]. One could hypothesize that, in individuals with prediabetes with low or low-normal thyroid function (i.e., high TSH and low FT4), lifestyle interventions or diabetes treatment could be prompted in an earlier phase than those with normal or high thyroid function. Alternatively, having prediabetes could be an argument to start treatment of subclinical hypothyroidism to aim for prevention of overt diabetes. Current guidelines do not recommend or specifically address screening of thyroid function or treatment of thyroid dysfunction in individuals with type 2 diabetes [31, 32].

The relative risk increase of developing diabetes with thyroid function differences is modest. However, due to the high population risk of diabetes, the implications on the absolute risk are large. Despite this high occurrence of both conditions in the general population, the relation between thyroid dysfunction and diabetes had remained largely unexplored. Further research is needed to determine to what extend the association could be driven by thyroid hormone-related acceleration of development of diabetes or perhaps by other mechanisms such as a common genetic predisposition. If our results are confirmed, subsequent studies could focus on screening and prevention strategies as well as questions concerning treatment of subclinical hypothyroidism in patients at risk for diabetes.

Strengths of our study include the large number of individuals, the variety of available confounders adjusted for, and the long follow-up. Furthermore, we were able to investigate both diabetes risk as well as progression from prediabetes to diabetes. Limitations of our study should also be acknowledged. Residual confounding cannot be excluded in an observational study, even with the large number of potential confounders adjusted for in our analyses. Furthermore, the Rotterdam Study is predominantly composed of white participants aged 45 years and older and results may therefore not be generalizable to other populations.

## Conclusions

In conclusion, our results suggest that low and low-normal thyroid function are related to an increased risk of diabetes. In individuals with prediabetes and low and low-normal thyroid function, the risk of progression to diabetes seems more prominent. Our data provide new insights into the magnitude of the risk of diabetes and prediabetes associated with variations of thyroid function within the normal range. More research is needed to confirm these current findings in various populations. Subsequent studies could address possible screening and treatment modalities for both diabetes and thyroid dysfunction.

## Additional files

Additional file 1: Table S1. Sensitivity analyses for association between thyroid function and risk of diabetes. (DOCX 19 kb) Additional file 2: Table S2. Association between thyroid function in normal range and the risk of incident diabetes in individuals with prediabetes. (DOCX 19 kb)

## Abbreviations

CI: Confidence interval
FT4: Free thyroxine
HR: Hazard ratio
RS: Rotterdam Study
TPOAb: Thyroid peroxidase antibodies
TSH: Thyroid-stimulating hormone
