# Supplementary material for: Thyroid function and risk of type 2 diabetes: a population-based prospective cohort study
Source: BMC Med. 2016 Sep 30;14:150. doi: 10.1186/s12916-016-0693-4 (PMC5043536; doi:10.1186/s12916-016-0693-4)
Supplement: Additional file 1: Table S1. — Sensitivity analyses for association between thyroid function and risk of diabetes. (DOCX 19 kb) [file 12916_2016_693_MOESM1_ESM.docx]

**Thyroid function and risk of type 2 diabetes: a population-based prospective cohort study**

**Supplemental data**

| **Supplemental Table 1. Sensitivity analyses for association between thyroid function and risk of diabetes** | | | | |
| --- | --- | --- | --- | --- |
| **Sensitivity analysis** | **HR (95% CI) Model 1** | **HR (95% CI) Model 2** | **Incident Diabetes** | **Total participants** |
| **Full range** |  |  |  |  |
| Excluding levothyroxine at baseline |  |  |  |  |
| TSH mIU/L | 1.12 (1.05-1.19) | 1.08 (1.02-1.16) | 772 | 8214 |
| Free T4 pmol/L | 0.95 (0.92-0.98) | 0.96 (0.92-0.99) | 771 | 8214 |
|  |  |  |  |  |
| Excluding thyroid function altering medication^a^ at baseline and follow-up |  |  |  |  |
| TSH mIU/L | 1.13 (1.05-1.17) | 1.09 (1.02-1.17) | 757 | 8107 |
| Free T4 pmol/L | 0.95 (0.92-0.98) | 0.96 (0.93-0.99) | 756 | 8107 |
|  |  |  |  |  |
| **Normal range** |  |  |  |  |
| Excluding thyroid function altering medication^a^ at baseline and follow-up |  |  |  |  |
| TSH mIU/L | 1.16 (1.04-1.30) | 1.14 (1.02-1.27) | 672 | 7106 |
| Free T4 pmol/L | 0.93 (0.90-0.97) | 0.94 (0.91-0.98) | 672 | 7106 |
|  |  |  |  |  |
| Excluding thyroid function altering medication^a^ at baseline and follow-up and including only prediabetic individuals |  |  |  |  |
| TSH mIU/L | 1.26 (1.16-1.36) | 1.21 (1.04-1.41) | 352 | 1124 |
| Free T4 pmol/L | 0.90 (0.85-0.95) | 0.92 (0.86-0.97) | 352 | 1124 |
| Model 1: adjusted for sex, age, smoking, fasting serum glucose levels and cohort. Model 2: adjusted for sex, age, smoking, cohort, fasting serum glucose levels, fasting serum insulin levels, systolic blood pressure, diastolic blood pressure, blood pressure lowering medication, HDL cholesterol and body-mass index. Normal range is defined by TSH 0.4-4.0 mIU/L and FT4 11-25 pmol/L. Thyroid function altering medication is defined as use of levothyroxine, anti-thyroid drugs, amiodarone, corticosteroids or iodine.  Results are presented as HR per doubling of TSH on average and per one increase in pmol/L of FT4. Abbreviations: CI confidence interval, FT4 free thyroxine, HR hazard ratio, TSH thyroid-stimulating hormone. | | | | |
