# Supplementary material for: Thyroid function and risk of type 2 diabetes: a population-based prospective cohort study
Source: BMC Med. 2016 Sep 30;14:150. doi: 10.1186/s12916-016-0693-4 (PMC5043536; doi:10.1186/s12916-016-0693-4)
Supplement: Additional file 2: Table S2. — Association between thyroid function in normal range and the risk of incident diabetes in individuals with prediabetes. (DOCX 19 kb) [file 12916_2016_693_MOESM2_ESM.docx]

**Thyroid function and risk of type 2 diabetes: a population-based prospective cohort study**

**Supplemental data**

| **Supplemental Table 2. Association between thyroid function in normal range and the risk of incident diabetes in individuals with prediabetes** | | | | | |
| --- | --- | --- | --- | --- | --- |
| **Thyroid function measurements** | **HR (95% CI) Model 1** | **HR (95% CI) Model 2** | **Absolute risk** | **Incident Cases** | **Total participants** |
|  |  |  |  |  |  |
| Tertiles of TSH |  |  |  |  |  |
| 0.40-1.44 mIU/L | REFERENCE | REFERENCE | 23.7% | 95 | 378 |
| 1.44-2.19 mIU/L | 1.48 (1.13-1.93) | 1.47 (1.13-1.92) | 27.7% | 129 | 379 |
| 2.19-3.97 mIU/L | 1.44 (1.11-1.88) | 1.37 (1.05-1.78) | 32.2% | 134 | 380 |
| *P for trend* | 0.004 | 0.015 |  |  |  |
|  |  |  |  |  |  |
| Tertiles of FT4 |  |  |  |  |  |
| 11.06-14.79 pmol/L | REFERENCE | REFERENCE | 32.8% | 132 | 379 |
| 14.79-16.57 pmol/L | 0.85 (0.66-1.09) | 0.87 (0.68-1.12) | 28.0% | 126 | 378 |
| 16.57-23.29 pmol/L | 0.63 (0.48-0.82) | 0.70 (0.53-0.92) | 23.2% | 100 | 380 |
| *P for trend* | < 0.001 | 0.002 |  |  |  |
| Model 1: adjusted for sex, age, smoking, fasting serum glucose levels and cohort. Model 2: adjusted for sex, age, smoking, cohort, fasting serum glucose levels, fasting serum insulin measurements, systolic blood pressure, diastolic blood pressure, blood pressure lowering medication, cholesterol and body-mass index. Normal range of TSH is defined by 0.4-4.0 mIU/L and normal range FT4 is defined by 11-25 pmol/L and participants not using levothyroxine. Absolute risk estimates were calculated for a follow-up time of 7 years adjusted for the covariates of Model 2 of the Cox-model.  Abbreviations: CI confidence interval, FT4 free thyroxine, HR hazard ratio, TSH thyroid-stimulating hormone. | | | | | |
